# Detecting ovarian cancer in primary care: can we do better?

**DOI:** 10.3399/bjgp22X719825

**Published:** 2022-07-01

**Authors:** Garth Funston, Emma J Crosbie, Willie Hamilton, Fiona M Walter

**Affiliations:** Primary Care Unit, Department of Public Health and Primary care, University of Cambridge, Cambridge.; Gynaecological Oncology Research Group, Division of Cancer Sciences, University of Manchester, Manchester.; University of Exeter Medical School, University of Exeter, Exeter.; Wolfson Institute of Population Health, Faculty of Medicine & Dentistry, Queen Mary University of London, London.

The NHS Long Term Plan sets a target of detecting three-quarters of cancers at an early stage (I–II) by 2028.^1^ Currently, fewer than half of UK women with ovarian cancer are diagnosed at an early stage.

In 2021 the results of the UK Collaborative Trial of Ovarian Cancer Screening (UKCTOCS) were published.^2^ Disappointingly, this landmark trial showed no reduction in mortality through screening. Even if a promising new screening approach were developed today, the time needed to rigorously evaluate tests and pathways means we are unlikely to see an evidence-based ovarian cancer screening programme implemented within the next decade. In the absence of screening, most women will continue to be diagnosed following the onset of symptoms. Ensuring primary care assessment, triage, and specialist referral is as accurate and timely as possible is key to reducing diagnostic delay and optimising outcomes.

## THE CHALLENGE

Ovarian cancer is the sixth most common cancer and the sixth most common cause of cancer death in UK women.^3^ Most are not diagnosed until the cancer is advanced, which contributes to the poor outcomes, including the 5-year net survival of 43%.^4^ Earlier detection could enable more women to be diagnosed with more treatable disease.

Symptoms can occur in both early- and late-stage disease,^5^ but these are generally non-specific posing a diagnostic challenge. In the UK, one-third of women present to primary care with relevant symptoms three or more times before specialist referral, and one-quarter take ≥160 days to be diagnosed following presentation.^6^^,^^7^

## PRIMARY CARE TESTING APPROACHES

Given the non-specific nature of symptoms of possible ovarian cancer, triage tests are generally used to select patients for specialist investigation and/or referral; however, no international consensus exists on the most appropriate test or test sequence. Within the UK recommendations vary, with the National Institute for Health and Care Excellence (NICE) advocating a sequential approach of cancer antigen 125 (CA125) testing followed by transvaginal ultrasound (TUS) only if the CA125 is ≥35 U/ml,^8^ while Scottish guidelines recommend requesting the tests in parallel with an urgent referral to gynaecology if either is abnormal.^9^

Despite being recommended as the first-line investigation for suspected ovarian cancer by NICE in 2011, the performance of CA125 in primary care has only recently been elucidated. In a study using routinely collected data from over 50 000 CA125-tested women, CA125 had a sensitivity of 77% for all ovarian cancers and 85% for invasive subtypes (those responsible for most ovarian cancer mortality).^10^ So, while CA125 detects most ovarian cancers when used in primary care, a significant proportion of women have levels below the recommended cut-off and, under NICE guidelines, would not undergo further investigation for ovarian cancer. Women with CA125 levels <35 U/ml take around twice as long to be diagnosed with ovarian cancer as women with CA125 levels ≥35 U/ml, but are more frequently diagnosed with early-stage disease, which may be due in part to differences in cancer behaviour.^11^

No large studies have evaluated TUS for ovarian cancer detection in primary care, so its diagnostic performance in this setting remains unknown.

## NATIONAL GUIDELINES

The new NHS England Faster Diagnostic Standard set a target that cancer should be diagnosed (or refuted) within 28 days from GP referral.^1^ In 2021 a pathway for gynaecological cancer was developed to help meet this target, introducing improvements such as secondary care ‘one-stop clinics’.^12^ These guidelines may shorten intervals in secondary care but fail to consider the primary care component of the diagnostic pathway.

The NICE primary care ovarian cancer pathway was developed over a decade ago. Recent evidence indicates the ovarian cancer pre-test probability is four times higher, and the positive predictive value of CA125 (at the ≥35 U/ml cut-off) 12 times higher than the estimates used to inform NICE recommendations.^8^^,^^10^ Given these much higher risk estimates, a lower CA125 cut-off and less stringent criteria for 2-week wait (2WW) referral may be appropriate, subject to an updated health economic assessment.

## IMPROVING THE PATHWAY: OPTIONS?

Promising new ovarian cancer specific tests and multi-cancer early detection panels are being evaluated, but none are ready for implementation within primary care. Nevertheless, different approaches could be adopted using existing tests, which may expedite ovarian cancer diagnosis.

### Increasing imaging capacity

The majority of GPs in one pre-COVID-19 survey reported that it took more than 2 weeks from requesting a TUS to receiving the result, even if requested as ‘urgent’.^13^ It has been estimated that a 3-month delay in ovarian cancer diagnosis would reduce 10-year net survival by 7%–18% for women with the disease, depending on age.^14^ This highlights the importance of addressing sources of delay within the pathway such as limited access to diagnostic tests.

Encouragingly, the NHS plans to expand GP access to imaging through ‘community diagnostic hubs’.^1^ To enable accurate detection of suspicious ovarian pathology, it is important not only that equipment is available but that there are sufficient radiographers with expertise in ovarian cancer imaging. The new pathway for gynaecological cancer recommends that if a TUS is abnormal then the woman should be referred directly to secondary care,^12^ which should help minimise delay within the pathway.

### Parallel testing

**Figure 1.**
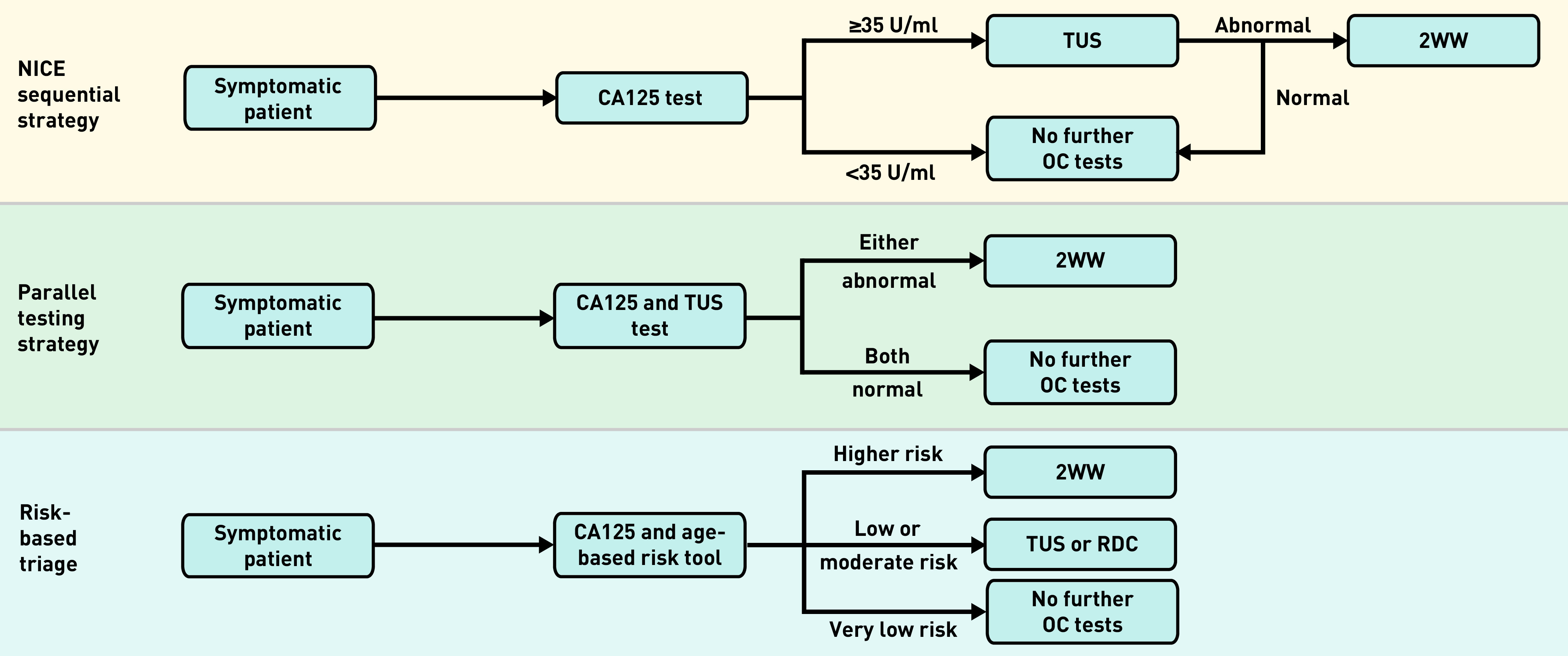
**Comparison of National Institute for Health and Care Excellence (NICE) advocated sequential testing strategy with parallel testing and individual risk-based triage strategies. 2WW = 2-week wait. CA125 = cancer antigen 125. OC = ovarian cancer. RDC = rapid diagnostic centre. TUS = transvaginal ultrasound.**

Although CA125 and TUS have never been compared in primary care, in secondary care studies some women with ovarian cancer and CA125 levels <35 U/ml have an abnormal TUS.^8^ Performing these tests in parallel rather than sequentially in primary care, with an abnormal result in either test triggering a 2WW referral (Figure 1), should improve the sensitivity of the primary care testing strategy, and may shorten primary care intervals. The potential for earlier diagnosis must be balanced against potential harms, as a greater number of women (most of whom would not have cancer) would be referred via the 2WW pathway, some of whom will undergo computed tomography (CT), and thus radiation exposure, and/or invasive tests. Given the current limitations on TUS access, and COVID-19-related pressure on cancer services contributing to some of the longest waiting times on record, any move to a dual testing approach would ideally be accompanied by an increase in capacity.

### Individual risk-based triage

The risk of a women having ovarian cancer varies by age and CA125 level; using a single CA125 cut-off is an oversimplification.^10^ Published models provide the probability of cancer based on age and CA125 level.^10^ This information could be provided alongside blood test results to GPs and used to select those at higher risk (NICE 3% specialist cancer investigation risk-threshold) for a 2WW referral, those at ‘low but not no risk’ (1%–2% cancer risk) for community TUS or referral to a rapid diagnostic centre (RDC), while affording those at very low risk (majority of CA125-tested patients) reassurance. As a significant proportion of women with high CA125 levels have other cancers — 20% of women aged ≥50 years with a CA125 ≥35 U/ml^10^ — referral to a RDC, where investigations are performed for multiple cancers simultaneously, could facilitate earlier cancer diagnosis.

## SUMMARY

While new standards and guidelines have been developed to reduce waiting times in secondary care, timely diagnosis depends on the pathway as a whole. Ensuring that we are using the best available tests in the best possible way, and that tests are readily accessible in primary care, has a central role to play in driving earlier ovarian cancer detection. In light of new evidence, a review of national guidelines should now be prioritised.
